# Cyclophilin Inhibitors as a Novel HCV Therapy

**DOI:** 10.3390/v2081621

**Published:** 2010-08-05

**Authors:** Hengli Tang

**Affiliations:** Department of Biological Science, Florida State University, Tallahassee, FL 32306-4295, USA; E-Mail: tang@bio.fsu.edu; Tel.: +1-850-645-2402; Fax: +1-850-645-8447

**Keywords:** Cyclophilins, Cyclosporine A, immunosuppression, nonstructural protein 5A/5B, HCV replication

## Abstract

A critical role of Cyclophilins, mostly Cyclophilin A (CyPA), in the replication of HCV is supported by a growing body of *in vitro* and *in vivo* evidence. CyPA probably interacts directly with nonstructural protein 5A to exert its effect, through its peptidyl-prolyl isomerase activity, on maintaining the proper structure and function of the HCV replicase. The major proline substrates are located in domain II of NS5A, centered around a “DY” dipeptide motif that regulates CyPA dependence and CsA resistance. Importantly, Cyclosporine A derivatives that lack immunosuppressive function efficiently block the CyPA-NS5A interaction and inhibit HCV in cell culture, an animal model, and human trials. Given the high genetic barrier to development of resistance and the distinctness of their mechanism from that of either the current standard of care or any specifically targeted antiviral therapy for HCV (STAT-C), CyP inhibitors hold promise as a novel class of anti-HCV therapy.

## 1. Introduction

Like all obligate intracellular parasites, viruses depend upon their host for survival and reproduction. Certain host proteins therefore assume, in addition to their normal cellular function, the unbecoming title of “cellular cofactors” for viral infection. In the majority of cases these proteins are simply too important to the host to be considered practical drug targets. Occasionally though, an opportunity for targeting a cellular cofactor would emerge, most often as a result of functional redundancy at the cellular but not the viral level. For RNA viruses with high mutation rates and great diversity, directing intervention at a cellular cofactor may offer the advantage of higher genetic barrier to development of resistance. A prominent success story for this strategy is the FDA approval of an anti-HIV drug that targets the C-C chemokine receptor type 5 (CCR5) [1].

Hepatitis C virus (HCV) infection tends to become chronic in most patients and can lead to liver fibrosis, cirrhosis, and hepatocellular carcinoma. The current treatment of HCV infection is not optimal, mainly as a result of resistance to interferon (IFN) in a large percentage of patients, especially those infected with genotype 1. New classes of anti-HCV drugs are being pursued by virtually all major pharmaceutical companies. In addition to the specifically targeted antiviral therapy for HCV (STAT-C), whose target is viral proteins, small-molecule compounds that inhibit an essential cellular cofactor, Cyclophilin A (CyPA), have also generated considerable interests because of their promising efficacy results in both preclinical and clinical studies. Here I summarize current understanding of CyPA’s role in the HCV life cycle and progress of the preclinical and clinical development of CyP inhibitors (CPIs) as anti-HCV drug candidates. I will also discuss potential mechanisms of resistance to CPIs, on the basis of research carried out in cell-culture systems.

## 2. Cyclosporine A and HCV infection

Cyclophilins are high-affinity protein ligands of Cyclosporine A (CsA), a compound originally isolated from the soil fungus *Tolypocladium inflatum* and an immunosuppressive and anti-inflammatory drug approved for use in organ-transplant patients. Another commonly used immunosuppressive compound, FK506 (Tacrolimus), binds to a different group of proteins, called FK506-binding proteins. Despite having distinct individual structures for both the compounds and the proteins, the CsA-CyPA complex and that of FK506 with the FK506-binding proteins, through a composite surface, bind to a common target, calcineurin, and block its phosphatase activity, which is critical for the expression of the cytokine T-cell activation [2]. The earliest indication that CsA might suppress HCV replication was reported even before the virus was cloned and named as HCV. In two experimentally infected chimpanzees, the histometric scores representing ultrastructural changes in hepatocytes improved with intravenous administration of CsA [3]. The authors speculated that CsA had inhibited the proliferation of HCV, which was still identified as non-A, non-B type 1 hepatitis virus (NANBHV) [4]. Fifteen years later, CsA was demonstrated to inhibit HCV replication directly in a cell culture–based replicon system [5,6]. The finding that CsA’s anti-HCV effect was independent of its immunosuppressive function was promising, as the idea of treating a viral infection with an immunosuppressive drug seemed counterintuitive. In addition, CsA's inhibition of HCV through a mechanism distinct from that of IFN raised hope of a synergy with IFN in a combination treatment.

In HCV-infected liver-transplant patients, benefits of CsA over FK506, which does not inhibit HCV *in vitro*, in alleviating the severity of recurrent HCV have been supported by some studies but disputed by others [7–13]. On the other hand, combination of IFN and CsA was clearly more effective in achieving sustained virological response than was IFN monotherapy, especially in patients with genotype 1 virus, high viral load, or both [14,15]. More recently, several CsA derivatives that are devoid of immunusuppresive function have been demonstrated to be efficacious in inhibiting HCV replication in both preclinical and clinical studies [16–22]. The leading compound of this class, Debio-025, is currently being tested in a phase IIb human trial. A number of reasons may account for the failure of CsA to show any anti-HCV benefits in certain transplant patients. First, the immune functions of these patients are suppressed by CsA and the antiviral effect alone may not be enough to suppress the virus without the help of the immune system; second, the standard dosage of CsA used in these transplant patients may be low as far as the anti-HCV function is concerned. Debio-025, which is already more potent than CsA at inhibiting HCV replication *in vitro,* was given at 600–1200 mg BID, whereas the daily dosage of CsA in the liver-transplant patients was 4–5 times lower.

## 3. Cyclophilin A as an essential HCV cofactor

*In vitro* studies of CsA derivatives revealed a correlation between viral inhibition and CyP binding [20,23], suggesting that one or more CyPs are the direct targets of CsA’s inhibitory action on HCV replication. Despite early suggestions that CyPB and/or CyPC plays an important role in HCV replication and that different genotypes of HCV require different CyPs [23,24], results from the author’s lab first indicated a universal and acute requirement for CyPA, but not CyPB or CyPC, for the replication of genotypes 1a, 1b, and 2a [25]. That CyPA is the most important of the CyP isoforms for HCV replication was quickly confirmed by more independent studies [26–29]. Considering the drastic difference (at least 10-fold) between the expression level of CyPA and that of the other CyP isoforms in liver cells [25], it is perhaps not surprising that the most abundant form, CyPA, is also the most critical for HCV replication. Previous work has shown that, although both CyPA and CyPB can bind to HIV Gag protein *in vitro* [30], knocking out CyPA alone in a T-cell line was able to completely block infection by HIV [31], which also requires CyPA as an essential cofactor to infect these cells. Note that other CyPs, having structures highly similar to that of CyPA [32–34], are probably able to bind to HCV protein *in vitro* when large amounts of recombinant CyP proteins are used [23,35–37]. Correspondingly, these additional CyP isoforms may contribute to HCV replication either when they are overexpressed or when CyPA level is reduced by RNA interference.

Following the identification of CyPA as a high-affinity intracellular ligand for CsA by Handschumacher and colleagues [38], two independent studies assigned the peptidyl-prolyl isomerase (PPIase) activity that they were tracking to CyPA by purifying the *in vitro* activity that catalyzed the *cis-trans* isomerization of Xaa-Pro amide bonds [39,40]. Interestingly, although CsA-binding potently inhibits the PPIase activity of CyPA, the isomerase activity appears to be dispensable for calcineurin binding and immune suppression. Several point mutations (R55A, F60A, and H126Q) in the active site of CyPA reduced PPIase activity by 190- to 1000-fold on a peptide substrate, yet retained CsA-binding and calcineurin inhibition [41]. The same mutations have also been shown to be crippling for CyPA’s HCV cofactor function in cDNA rescue experiments [26,27,42], suggesting that the PPIase activity is required for HCV replication.

For HIV infection, CyPA recognizes a single proline residue on the capsid protein to which it binds in a CsA-sensitive manner [43], but the potential target of CyPA’s PPIase activity relevant for HCV replication is probably not restricted to a particular proline. In fact, several HCV proteins (NS5A, NS5B, and NS2) have been suggested to be targets of CyPA’s cofactor function [23,28,35,37,44]. In particular, a CsA-sensitive interaction between NS5A and CyPA can be demonstrated *in vitro* [44–46], and the CyPA binding site has been mapped, at least in part, to the proline-rich domain II of NS5A. Nuclear magnetic resonance analysis revealed that even within domain II, multiple prolines are likely to be involved in CyPA binding and isomerization [44].

Precisely why HCV needs CyPA to replicate has not yet been resolved. One proposed function of CyPA is to ensure the integrity of the viral replication complexes (RC) [42]. Although the majority of the CyPA protein is found to be cytosolic, a small percentage cofractionates with intracellular membranes. CsA treatment disrupts this membrane association of CyPA [42]. In addition, a loss of membrane-protected and protease-resistant NS5B can be observed in replicon cells treated with CsA, suggesting CyPA’s action, either through NS5A or directly, is required for the proper incorporation of NS5B into the membrane-enclosed RC. A subsequent study independently confirmed the CsA-sensitive association of CyPA with cellular membranes but argued against a role for CyPA in the RC incorporation of NS5A or NS5B [47]. Understanding more details of the RC assembly process and the mechanism of selective incorporation of only a small percentage of NS proteins [48] might require a biochemically traceable *in vitro* replication assay.

## 4. Inhibition of HCV replication by cyclophilin inhibitors

**Figure 1.**
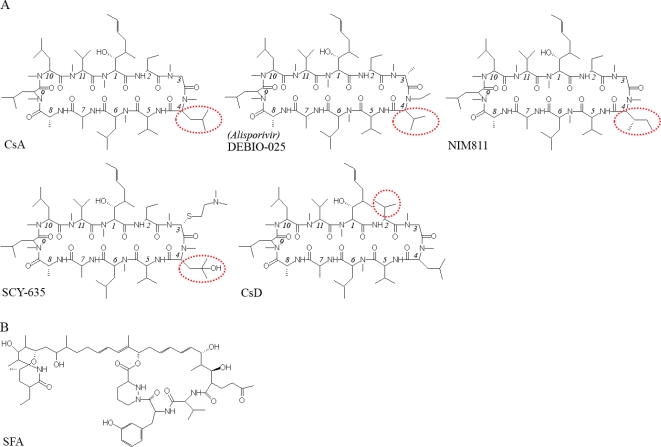
Structures of CPIs that inhibit HCV replication. (A) CsA derivatives that lack the immunosuppressive function. (B) Sanglifehrin A, a CyP-binder with a structure unrelated to CsA.

CsA derivatives that lack the immunosuppressive function have been shown to inhibit HCV replication in cell-culture models [16,20,22,24,49]. The structures of four such compounds, Debio-025, NIM 811, SCY-635, and CsD, are shown in Figure 1A. The CyP-binding surface of CsA, made up of residues 1, 2, 3, 9, 10, and 11, appears to conserved, as all the derivatives can bind CyPs and inhibit their PPIase activity. On the other hand, none of these derivatives could form a stable tertiary complex with CyPA and calcineurin, as CsA can; this explains their inability to suppress T-cell activation and IL-2 production. Residues 4 through 7 of CsA are important for calcineurin binding [2]. In particular, the N-methyl-Leu side chain at position 4 of CsA appears to be critical, as it occupies the hydrophobic pocket of the calcineurin protein [50]. With the exception of CsD, the CsA derivatives all differ from CsA and from each other at position 4. The N-ethyl-Val of Debio-025 is too short to enter the calcineurin pocket [50], and the side-chain modifications on NIM811 (in which a sec-butyl replaces an isobutyl group) and SCY-635 (in which a hydroxyl is substituted at the γ-carbon of N-methyl-Leu) probably affect calcineurin binding through steric hindrance. The reason for the reduced immunosuppressive function of CsD is not yet understood [51].

Debio-025 (also named Alisporivir) is a synthetic derivative of CsA that has chemical modifications at positions 3 and 4. These changes appear not only to abolish its immunosuppressive function but also to increase its inhibitory effect on CyP, as suggested by an approximately 10-fold lower EC_50_ against HCV compared to that of CsA [16]. The anti-HCV activity of Debio-025 has been extensively studied in cell culture [16], mice [17], and humans [19,52]. In cell-culture systems, Debio-025 efficiently inhibited replication of both subgenomic replicons and a full-length infectious virus; it was also able to clear replicon cells of persistent replication [18], much the same way interferon did in these cells [53]. In a human liver SCID/uPA mouse model, where the mouse liver has been repopulated by transplanted human hepatocytes that can then be infected with HCV patient serum [54], the combination of Debio-025 (100 mg/kg/day, orally) with pegylated interferon-α (peg-IFN) (30 μg/kg subcutaneously twice weekly) resulted in 100-fold decrease in serum HCV RNA levels while peg-IFN alone only resulted in a 10-fold decrease [17]. Similar results were obtained with chimeric mice infected with either genotype (GT) 1a or 1b HCV. Curiously, Debio-025 alone had no effect on HCV replication in this setting. In addition, because the mice that received the combination of CsA (100 mg/kg/day orally) and peg-IFN all died with 4 days of the treatment, a direct comparison of Debio-025 and CsA in this model was not available.

In human trials, the antiviral effect of Debio-025 was first evaluated in a small group of HIV/HCV-infected patients as a monotherapy [52]. The 19 co-infected patients were divided into a placebo group (n = 3) and a Debio-025 group (n = 16) which received 1200 mg of Debio-025 twice daily for 14 days. At the end of the study, the mean HCV viral load was decreased by 3.6 logs in the Debio-025 group but only by 0.7 log in the placebo group (p < 0.0001). The anti-HIV effect was less impressive with a 1.1-log decrease of HIV RNA copies/mL in the Debio-025 group. With the control group also showing a 0.5-log reduction, no significant difference between compound and placebo could be demonstrated in terms of HIV inhibition in this limited trial. Given the promising results regarding HCV infection, however, a larger, phase IIa clinical trial was then carried out to determine the efficacy and safety of Debio-025 in combination with peg-IFNα-2a [19]. Ninety patients were randomly divided into five groups and treated for 29 days with the following regimens: peg-IFN with placebo; peg-IFN with 200 mg/day Debio-025; peg-IFN with 600 mg/day Debio-025; peg-IFN with 1000 mg/day Debio-025; and 1000 mg/day Debio-025 only. The two combination groups with higher Debio-025 concentrations achieved significantly greater reduction of HCV RNA (4.6 logs for the 600-mg combination and 4.8 logs for the 1000-mg combination) than either of the monotherapies (2.5 logs for peg-IFN monotherapy and 2.2 logs for the Debio-025 monotherapy). In addition, viral load was undetectable in 66% of the patients in the 1000-mg combination group but in only 25% of the patients treated with either of the monotherapies. Currently, a phase IIb trial is underway that will examine the safety and efficacy of Debio-025 in combination with the standard of care for HCV: peg-IFNα plus ribavirin. The study is being conducted in treatment-naïve patients infected by the most prevalent genotype (GT1), and results of the trial are not yet available as of this writing.

NIM811 was the first CsA derivative lacking immunosuppressive function to be synthesized, albeit as an inhibitor for HIV infection [55]. Like that of Debio-025, the higher CyP-binding affinity of NIM811 [49] is correlated with a more potent anti-HCV activity in the replicon system [20]. Importantly, combining NIM811 with NS3-4A protease or NS5B polymerase inhibitors not only resulted in an additive inhibition of viral replication but also suppressed the emergence of resistance to the viral enzyme inhibitors [21].

SCY-635 is another CsA-based CyP inhibitor with modifications at positions 3 and 4 [22]. It inhibited the PPIase activity of CyPA *in vitro* and suppressed HCV replication in replicon cells. Importantly, SCY-635 exhibited no inhibition of calcineurin phosphatase activity when complexed with CyPA at 2 μM, a concentration 20 times the EC_50_ for replicon inhibition *in vitro*. In a phase 1b monotherapy trial of GT1-infected patients, the treatment groups were given daily doses of 300, 600, and 900 mg for 15 days, either once daily or in divided doses three times per day. The highest dose of SCY-635 produced significant antiviral activity and resulted in a viral load reduction of 2.2 logs. Of the 20 subjects who were given SCY-635 three times daily, one achieved undetectable RNA levels at day 15 [56].

CsD is a cyclosporine analogue that has a valine in the place of an aminobutyric acid at position 2 of CsA [57]. Although it could inhibit HCV replicon *in vitro* [24], it does not appear to be as potent as the other derivatives and may have retained part of CsA’s immunosuppressive function [51].

In addition to CsA-derived inhibitors, CyPA inhibitors that are structurally distinct from CsA, such as the macrocyclic compound Sanglifehrin A (SFA) produced by the actinomycetes strain *Streptomyces* A92-308110 (Figure 1b), can also suppress HCV replication [58,59], further validating the critical role of CyPA in HCV life cycle. The immunosuppressive function of Sanglifehrin A, which appears unrelated to calcineurin inhibition [60,61], will likely prevent the direct use of the unmodified compound as an anti-HCV drug. Nevertheless, these data suggest the feasibility of developing chemically independent CyPA inhibitors for HCV suppression [59].

## 5. Viral resistance to CPIs

One of the theoretical advantages of targeting host cofactors rather than viral agents is the presumed higher genetic barrier to development of resistance. Mathematic modeling of viral dynamics suggests that both single and double mutations exist in the HCV population before treatment and that at least one more mutation is likely to emerge during treatment [62]. On the basis of this calculation, for small molecule inhibitors whose targets are viral enzymes such as NS3/4A protease or the NS5B polymerase, resistant viruses are expected to arise rapidly during therapy unless combination therapies with genetic barriers of four or more mutations are used. Several labs have characterized HCV resistance to CsA *in vitro,* using either subgenomic replicons [26,35,37,58,59] or, in a more recent study, JFH-1 full-length virus [63]. Taking advantage of a sortable replicon that contains a GFP reporter inserted into the NS5A gene [64,65], Robida *et al.* used antibiotic selection and live cell sorting to isolate replicon cells that were approximately 17-fold more resistant to CsA than genotype- and reporter-matched wild-type replicon. As expected, these cells were also cross-resistant to Debio-025 and NIM 811 (Tang H, unpublished results). Because these compounds inhibit a cellular target, determining the relative contributions of cellular and viral mutations to the observed drug resistance exhibited by the selected replicon cells was important. By separating viral RNA and host cells of the resistant cell line and remixing them with naïve cells and wild-type replicon RNA, respectively [37,66], these authors demonstrated that mutations in viral RNAs, but not changes in the host cell, are responsible for CsA resistance.

Mutations in both NS5B and NS5A appear to contribute to CsA resistance, and early mapping studies generated a rather scattered mutation profile [26,35,37], suggesting that mutations in more than one site or even in more than one protein may be necessary to confer the full-level of resistance conferred by the selected replicon. Although this idea is consistent with a high genetic barrier to resistance, as supported by the lack of *in vivo* resistance so far, more recent results do point to a specific “Asp-Tyr” dipeptide motif (D320-Y321 in GT1 or D316-Y317 in GT2a) in the domain II of NS5A as a major regulator of HCV’s susceptibility to CsA and other CPIs, at least *in vitro* [45,58,59,63,67]. Mutation of Aspartate into Glutamate or of Tyrosine into Asparagine individually conferred CsA resistance in replicons of various genotypes, and the combination the D316E and Y317N mutations in a J6/JFH-1 full-length genome resulted in a virus that replicated several fold better in CyPA-knockdown cells than in control cells [63]. These results, together with the biophysical [44] and functional [63] mapping of the proline substrates to this segment of domain II, strongly support a major role of DY motif–containing peptide in determining HCV’s susceptibility to CPIs. Interestingly, although Sanglifehrin A treatment also selected for the D320E mutation [59], the highly potent NS5A inhibitor BMS-790052 selected for resistance mutations in the domain I of NS5A [68], suggesting that the BMS compound and CPIs use distinct mechanisms to inhibit NS5A function.

CyPA knockdown with RNA interference suppressed HCV infection, confirming that CsA blocks a critical function of CyPA itself, not a downstream effecter of the CsA-CyPA complex, as in the case of T cell suppression. In addition, the resistance to CsA was correlated with resistance to CyPA knockdown by shRNA [25], further indicating that HCV susceptibility to CsA is mediated by CyPA. Of note, regardless of the mutations identified, all the CsA-resistant replicons isolated are still inhibited by CsA at high concentrations (>4 μM), suggesting that the resistance was a result of reduced dependence on CyP rather than of a complete independence. Strong support for this idea was first demonstrated in an experiment where a CsA-resistant replicon was treated with both a shRNA directed at CyPA and 0.5 μM of CsA, to either of which the replicon was normally resistant. The heightened sensitivity (*i.e.*, disappearance of resistance) to the double treatment is best explained if the resistant replicon still required CyP, albeit at a much lower level, to replicate [25]. Conversely, overexpression of CyPA in replicon cells was shown to be correlated with reduced CsA sensitivity [26].

Level of sensitivity to CsA treatment also appear to vary depending upon the genotypes or forms of viral genomes used in the *in vitro* studies. For example, a JFH-1 based replicon was less sensitive to CsA than were GT1b replicons [69], and a chimeric replicon with JFH-1 NS5B inserted into a GT1b replicon backbone was more resistant to CsA treatment [29]. These results may have implications for clinical-trial designs if natural polymorphism can affect the effectiveness of CPI therapy, although there is currently insufficient patient data to indicate that this would be the case. Full-length JFH-1 virus has also been observed to be significantly (>100-fold) more sensitive to CsA and its derivatives than the corresponding NS3-NS5B subgenomic replicon. Two distinct, but not mutually exclusive, hypotheses have been proposed to explain this difference. Incorporation of NS2 into the subgenomic replicon may increase CsA sensitivity through an unidentified mechanism (no interaction between NS2 and CyPA has been demonstrated), or CyPA may play a role in an additional step in the HCV life cycle (e.g., assembly) that cannot be measured in the replicon system [26,28]. Interestingly, the NS5A mutations (D316E and Y317N) identified in the CsA-resistant full-length virus also conferred resistance in the NS3-NS5B and NS2-NS5B replicons, pointing to a role of NS5A as the “master regulator” of CsA susceptibility.

## 6. Potential mechanism of resistance to CPIs

How do mutations in the HCV genome confer reduced dependence on CyPA? Multiple mechanisms may be involved. The D316/320E and Y317/320N mutations in domain II of NS5A do not appear to alter the CsA sensitivity of the NS5A-CyPA interaction [45,46,63]. They do, however, cause a conformation change at a putative CyPA substrate site that could either increase the isomerization efficiency of the surrounding prolines or render the structure more similar to the product of the isomerization [63]. In either case, the mutant NS5A would require less CyPA to fold and/or function properly. A distinct set of NS5A mutations that is associated with CsA resistance has been identified by Kaul *et al.* to locate to the C-terminus of NS5A, near the cleavage site of the NS5A-5B precursor protein [26]. These authors observed a correlation between a delay in NS5A-5B cleavage and CsA resistance for the mutations. It was then hypothesized that CyPA-binding by NS5A is only possible during a short window before or immediately after NS5A-5B cleavage, which normally occurs rapidly. Delay in NS5A-5B cleavage by mutations would then allow more time for CyPA-binding, reducing CyPA dependence and conferring CsA-resistance. Finally, mutations in NS5B may increase the template-binding activity and RC incorporation of the polymerase, which probably depends on the efficient and proper folding of its viral cofactor, NS5A [42,67]. Although no structural information is available regarding any of the putative complexes of CyPA-NS5A, CyPA-NS5B, or CyPA-NS5A-NS5B, all the data so far are consistent with a role of CyPA in the proper assembly and function of the HCV replicase complex [42,70].

## 7. Summary

CyPA plays an indispensible role in the HCV life cycle, as evidenced by both chemical inhibition and genetic interference experiments in cell culture and *in vivo.* On the other hand, CyP inhibition appears to have little effect on cell survival, and CyPA-knockout in mice was well tolerated [71]. In addition, HCV resistance to CPIs appears to be relatively weak *in vitro* and rare *in vivo.* Accordingly, CPIs that lack immunosuppressive function hold promise as a new class of anti-HCV drugs that can be used either in combination with the current standard of care or with STAT-C therapies.
